# Use of the Satisfaction With Amplification in Daily Life Questionnaire to Assess Patient Satisfaction Following Remote Hearing Aid Adjustments (Telefitting)

**DOI:** 10.2196/medinform.2769

**Published:** 2014-09-02

**Authors:** Silvio Pires Penteado, Ricardo Ferreira Bento, Linamara Rizzo Battistella, Sara Manami Silva, Prasha Sooful

**Affiliations:** ^1^Medical SchoolOtorhinolaryngology DepartmentUniversity of Sao PauloSao PauloBrazil; ^2^Medical SchoolDepartment of Forensic Medicine, Medical Ethics and Medicine and Social WorkUniversity of Sao PauloSao PauloBrazil; ^3^ClinicAudiologyRoyal Darwin HospitalDarwinAustralia

**Keywords:** audiology, hearing aids, hearing loss, telemedicine, correction of hearing impairment, public policy, prosthesis fitting, telemedicine, questionnairies, quality improvement

## Abstract

**Background:**

Hearing loss can affect approximately 15% of the pediatric population and up to 40% of the adult population. The gold standard of treatment for hearing loss is amplification of hearing thresholds by means of a hearing aid instrument. A hearing aid is an electronic device equipped with a topology of only three major components of aggregate cost. The gold standard of hearing aid fittings is face-to-face appointments in hearing aid centers, clinics, or hospitals. Telefitting encompasses the programming and adjustments of hearing aid settings remotely. Fitting hearing aids remotely is a relatively simple procedure, using minimal computer hardware and Internet access.

**Objective:**

This project aimed to examine the feasibility and outcomes of remote hearing aid adjustments (telefitting) by assessing patient satisfaction via the Portuguese version of the Satisfaction With Amplification in Daily Life (SADL) questionnaire.

**Methods:**

The Brazilian Portuguese version of the SADL was used in this experimental research design. Participants were randomly selected through the Rehabilitation Clinical (Espaco Reouvir) of the Otorhinolaryngology Department Medical School University of Sao Paulo. Of the 8 participants in the study, 5 were female and 3 were male, with a mean age of 71.5 years. The design consisted of two face-to-face sessions performed within 15 working days of each other. The remote assistance took place 15 days later.

**Results:**

The average scores from this study are above the mean scores from the original SADL normative data. These indicate a high level of satisfaction in participants who were fitted remotely.

**Conclusions:**

The use of an evaluation questionnaire is a simple yet effective method to objectively assess the success of a remote fitting. Questionnaire outcomes can help hearing stakeholders improve the National Policy on Hearing Health Care in Brazil. The results of this project indicated that patient satisfaction levels of those fitted remotely were comparable to those fitted in the conventional manner, that is, face-to-face.

## Introduction

The prevalence of hearing loss in Brazil has been identified as 50%, specifically, individuals with permanent hearing loss of more than 41 decibels hearing level (dB HL) in the municipality of Minas Gerais [1]. If this rate were extrapolated to the total Brazilian population, a contingent of more than 9 million Brazilians would be identified as having permanent hearing impairment. Since 2004, Brazil’s public policy, National Hearing Health Care, has included the diagnosis, assessment, and rehabilitation of individuals with hearing loss. This policy culminated in the donation of hearing aids for Brazilian nationals seeking care at one of 139 centers accredited by the National Health System at various locations throughout the country. Many of the patients benefiting from this offer were from the adult and geriatric population. Patients were required to return to centers to receive necessary adjustments on their hearing aid settings and to receive information on care and usage of devices. The need for patients to return to the centers highlighted issues around transport to and from homes, cost of transport, as well as the need for carergivers to accompany patients.

The treatment of sensorineural hearing loss typically consists of the use of hearing aids (HA), and in cases of profound hearing loss, cochlear implants are used [2,3]. An HA is an electronic device that performs selective amplification, or amplifies signals at specific frequencies, using amplification strategies according to pathology and lifestyle of the hearing impaired [4]. The electronic architecture of a digital HA consists of a digital signal processor, microphone, and receiver [5].

**Figure 1:**
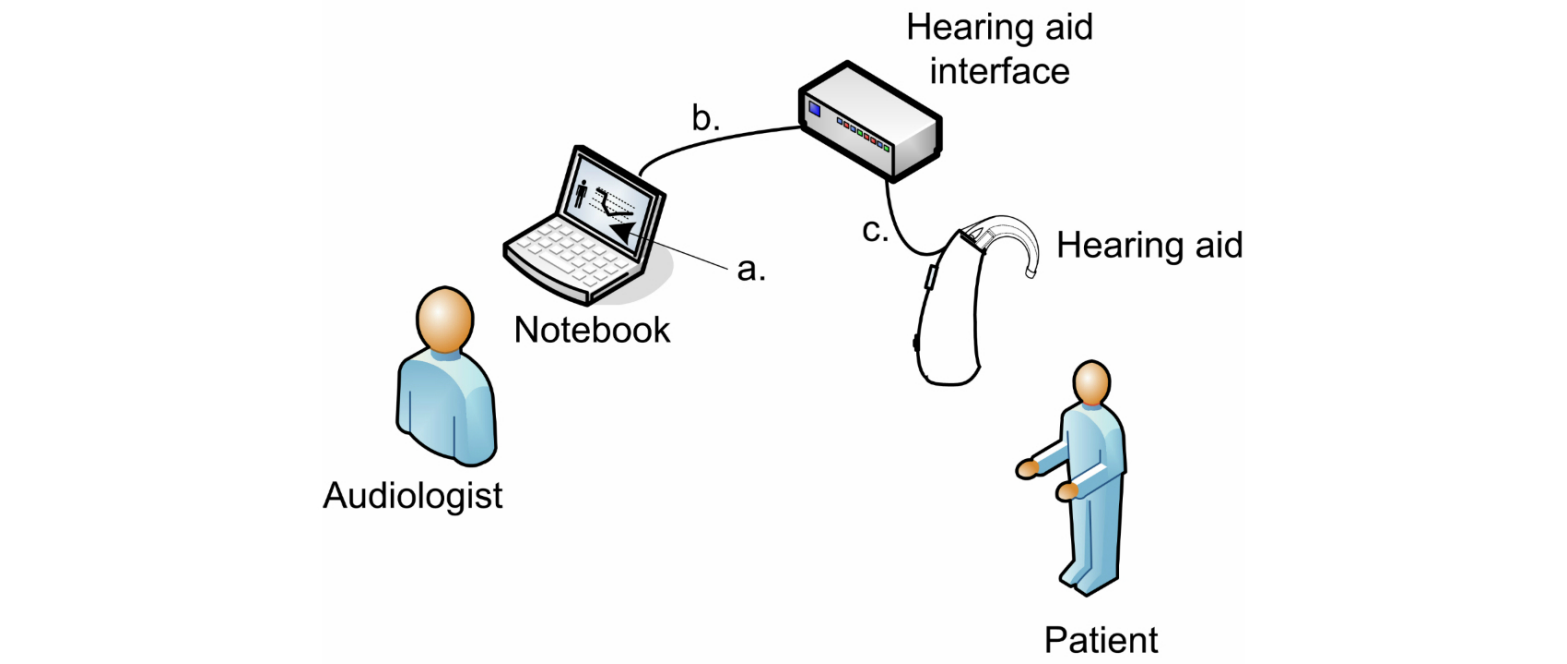
Diagram of a basic adjustment with digital HAs.

Fitting an HA demands specific technical knowledge of the electroacoustic characteristics of the device in relation to the patient’s hearing loss and, in Brazil, must be performed by an audiologist [6]. The fitting can be performed in a clinic or specialized hospital [7]. Digital HAs are fitted through an application known as a fitting program (Part a of Figure 1), which must be installed on a personal computer (PC). In this configuration (Figure 1), there must be a programming interface that connects the HA with the PC and the fitting application. A standardized programming interface commonly used is the HI-PRO device (GN ReSound A/S), which is connected to the PC via a universal serial bus (USB) cable (Part b of Figure 1). Finally, a programming cable is required to connect the HA to the interface (Part c of Figure 1).

After receiving an HA, it is essential to monitor the patient in order to understand changes that affect the auditory system [8] and thus support adjustments to HA settings while the patient acclimates. Subjective evaluation of the HA fitting can be accessed via patient questionnaires, such as the Satisfaction With Amplification in Daily Life (SADL), the International Outcome Inventory for Hearing Aids (IOI-HA), the Hearing Handicap Inventory for Adults, the Abbreviated Profile of Hearing Aid Benefit (APHAB), and the Hearing Inventory for Elderly (HHIE), among others.

Cox and Alexander [9] described the benefits of subjective self-assessment through patient questionnaires and scales. These reports yield valuable insights into the impact of impairment on everyday life and encourage the planning and execution of strategies to address the needs of the hearing impaired person. Additionally, self-reported outcome data can be used as a tool to validate the merit of a certain treatment program and can highlight areas of improvement. Aspects such as comfort, ability to hear with background noise, ease of hearing aid controls, ease of inserting and removing HAs, and so on, can be subjectively evaluated in the form of a satisfaction questionnaire. Objective assessment, however, requires the need for equipment and can be obtained by measuring the functional gain (the difference between the thresholds obtained with and without the HA in the free field mode), or by measuring the acoustic gain by using a probe microphone, known as Real Ear Insertion Gain.

Telemedicine has evolved in many health care areas. With a greater coverage area and lower operating costs, it is fast becoming a suitable method for assessment, diagnosis, and rehabilitation of various conditions. In an early work with hearing rehabilitation through telemedicine, Wesendahl [10] reported that remote fittings allow for experienced professionals to be present “in remote areas without restriction of time and geographic location” and that “telemedicine offers an opportunity to increase the efficiency of audiological methods and decrease expenses simultaneously”. Internationally, there have been many studies documenting the success of telemedicine by improving access to quality care [11,12]. In addition to hearing aids, cochlear implant remote mapping and adjustments [13-15] were highlighted by other studies [16,17]. Swanepoel et al [18] describes telemedicine: “although not the answer to all challenges related to global hearing loss, there is no alternative strategy that can offer the same positive impact on the current hearing loss burden in the near and foreseeable future.” A literature review of the demonstrated benefits of telemedicine in diverse areas of medicine can be found in Table 1 [19-38].

**Table 1:** Some publications in diverse areas of telemedicine.

Authors		Area of science	Region		Results/ remarks	
Chorbev & Mihajlov [19]		Several	Macedonia		Increased the population’s access to health services, reducing costs, spread of knowledge to more distant centers	
Jaakkola & Loula [20]		Public Policy	Finland		Decreased transport of patients and increased access to database of patients	
Khaleel et al [21]		Several	United States		Blood pressure, heart rate, body temperature, blood glucose and ECG signals can be transmitted to remote centers in real time	
Lavanya et al [22]		Dermatology	Singapore/United States		Dermatologists believed telemedicine to be beneficial when classroom visits were not possible or were troublesome	
Penzel et al [23]		Management	Germany/ France/ Portugal		It was possible to establish a European network of Internet access among different clinics and other partners	
Schreier et al [24]		Dermatology	Austria		Telemedicine using mobile phones equipped with camera enabled personalized therapy for psoriasis patients	
Siddiqua & Awal [25]		Several	Bangladesh		Telemedicine was considered a way to improve the quality of health services, with improved access and lower costs	
Shen et al [26]		Gynecology	United States		Preliminary studies have shown the effectiveness of developed systems, which improves the performance and diagnosis of breast diseases in remote areas	
Stoian et al [27]		Disaster management	Romania		Telemedicine provided immediate results with greater chances than traditional methods	
Sudhamony et al [28]		Oncology	India		Telemedicine offered great advantages in the practice of oncology as well as a decrease in the number of visits to emergency medical staff	
Arriaga et al [29]	Neurology			United States		Telemedicine is a viable delivery model for neurotology care delivery
Audebert et al [30]	Cardiology			Germany/United States		Telemedicine recommended for the treatment of stroke
Bonato [31]	Rehabilitation			United States		The emergence of new sensors attached to the body capture the activity level of patients, helping the effectiveness of pharmacological interventions more efficiently and specifically
Capampangan et al [32]	Vascular			United States		The hit rate for decision conduit thrombosis in patients with acute stroke was broader with the use of telemedicine than with the use of telephone
Cardoso et al [33]	Cardiology			Brazil		Public efforts are key to implementing remote distance interventions for underserved populations in Brazil
Knobloch et al [34]	Reconstructive surgery			Germany		Using phone with HD camera delivers positive results in reconstructive surgery
Levine & Gorman [35]	Neurology			United States		Use of computer-based technology may be integrated with the neuroradiology, among others, to take care to distant areas
Mora et al [36]	Surgery			United States		Solution-based telemedicine can help in intermittent surgical services among patients and medical professionals
Mucic [37]	Psychiatry			Denmark		Patients preferred and recommended the use of telepsychiatry instead of psychiatry face-to-face with interpreters
Sacco et al [38]	Neurology			Italy		Patients with subarachnoid hemorrhage require the implementation of telemedicine in rural areas to minimize the high incidence of mortality

With regard to telemedicine nationally, a study conducted by the University of Sao Paulo described the effectiveness of video conferencing for transmitting video-laryngoscopic images [39]. While in another study with 73 subjects [40], researchers found that teleaudiometry proved to be an efficient method of hearing screening, with results close to a sweep audiometry, and that the use of teleaudiometry could help identify cases of hearing loss, especially in cases of patients with poor access to professionals.

A study of remote HA fittings [41] listed the benefits perceived by patients as those of ease and convenience in the fitting process, as well as less travel time. These factors may increase the outcomes of successful fittings and reduce social stigma. Bento and Penteado [42] theorized that remote HA fittings also benefit health care staff with regard to reduced travel time and costs.

The National Policy on Hearing Health Care in Brazil was established through Ordinance #2.073/04 GM (September 2004). Ordinance #402 (February 2010) of the Ministry of Health established the program nationwide (ie, Brazil Telehealth). Telemedicine in a structured format has aimed to quantify how to expand and strengthen strategies in family health. The Brazilian Federal Board of Audiology issued Resolution #366 (April 2009) that defined the lawful exercise of Telehealth in audiology with the use of information technology in order to “assist, promote education and conduct health research”. According to official data, the government has become the largest purchaser of HAs in Brazil, as shown in Table 2.

**Table 2:** Investments in hearing health in Brazil (Ordinance #587 and #589).

Year	Total importation of HAs, units	Total purchases of HAs by the federal government, units	Percentage of purchases of HAs by the federal government, %
2005	169,575	113,983	67
2006	183,707	104,059	57
2007	214,310	134,194	57
2008	272,690	183,703	63
2009	280,578	184,646	66
2010	301,315	212,477	71
2011^a^	331,645	225,331	68
2012^a^	334,613	220,250	66
2013^a^	402,497	277,723	69

^a^Projection due to lack of official data.

Ordinance SAS/MS #58 specifies that HAs must be dispensed through centers accredited by the Unified Health System (SUS), where professionals must “perform diagnosis and rehabilitation of hearing loss in all age groups spanning neonates to geriatrics, and perform consulting ENT, neurological, pediatric audiological evaluation”. Additionally, they must “ensure rehabilitation through clinical treatment in otolaryngology; selection, fitting and provision of an HA and speech therapy”. The Ministry of Health has a list of 139 accredited centers to serve the population, which was 190,732,694 inhabitants divided into 8,514,876,599 km^2^, according to the 2010 census.

Our research describes a pilot study conducted with 8 patients fitted remotely through telemedicine, using the Brazilian Portuguese version of the SADL as a tool for measuring subjective satisfaction, with the goal of improving hearing health policies in Brazil.

## Methods

### Approval

This research protocol was approved by the Ethics Committee for Analysis of Research Projects under #0293/11. The data collection was completed between June and October 2012.

### Materials

The Brazilian Portuguese version of the SADL was used (see Multimedia Appendix 1); however, two SADL questions were discarded: #14: “Does the cost of your hearing aids seem reasonable to you?” and #15: “How pleased are you with the dependability (how often do they need repairs) of your hearing aids?” These deletions were justified because the participants did not buy their hearing aids and because it was not possible to evaluate the number of times the HA was sent out for repair, as only the initial and follow-up fitting appointments were conducted.

Additionally, Question 3 required a change as hearing aids were provided free of charge. The original question “Are you convinced that obtaining your hearing aids was in your best interests?” was changed to “Are you convinced that the received devices was the best option?” The documents used in this research are presented in Table 3.

**Table 3:** Study documents.

Document	Model	Version
Consent form	FMUSP	V 1.2
SADL questionnaire	Standard	Brazilian Portuguese
Terms and agreement of HA donation	TD	V 1.0

The Windows operating system was used for the data collection in this study because it has a large set of commercial applications available and is the largest PC platform used in Brazil. A broadband Internet connection was provided by the specialized unit (SU) and the remote unit (RU). The SU had a trained audiologist, who provided support and scientific training for the audiologist at the RU, thus acting as a facilitator.

The digital HAs donated and used this study were developed by researchers at the Medical School University of Sao Paulo, manufactured by Politec Saude (Multimedia Appendix 2). Only Behind-The-Ear (BTE) HAs were used in this study. A Portuguese version of the fitting application was supplied by the digital signal processor manufacturer ON Semiconductor. See Table 4 for the equipment we used and Table 5 for the applications used.

**Table 4:** Study equipment.

Description	Model	Manufacturer	Location
Notebook	Vostro 3500	Dell	SU
Notebook	Vostro 1510	Dell	RU
Hearing aid interface	HI-PRO	GN ReSound	RU
Router	78-0454ARB	GTS	SU
Router	ADSLCPE	ZTE	RU
Headphone	HT-301MV	Wasta	SU/RU
Web cam	1270	NA^a^	RU
Speakers	ND	FlexPc	RU

^a^Vostro 3500 Notebook has a built-in Web camera.

**Table 5:** Study applications and operating systems.

Name	Description	Version	Location
easyFIT	Hearing aids fitting	5.8.3.0	SU
TeamViewer	Remote access, VoIP^a^	7.0.14563	SU/RU
Medidor de velocidade de Internet	Internet speed meter on line	Full version	SU/RU
Operational system 32 bits	Windows 7	Professional	SU
Operational system 64 bits	Windows 7	Professional, Pack 3	RU

^a^VoIP: Voice over Internet Protocol. We chose VoIP by TeamViewer GmbH because it (1) had a free non-commercial version, (2) had compatible remote access, (3) allowed for message and file sharing, (3) allowed for recording sessions, (4) had a Portuguese version, (5) allowed adjustment of the microphone sensitivity, and (6) required minimal PC hardware requirements.

### Participants

Participants were randomly selected through the Rehabilitation Clinical (Espaco Reouvir) of the Otorhinolaryngology Department Medical School University of Sao Paulo based on the following criteria: (1) male and female individuals aged between 18 and 90 years, (2) with either no obstruction of the external auditory canal or middle ear pathology, or an absence of any neurological or psychological impairment, (3) individuals with no prior HA experience, (4) bilateral sensorineural hearing loss of varying degrees (ie, mild, moderate, moderate-severe), (5) postlingual hearing loss, and (6) native Brazilians. Table 6 shows a summary of the participants in the study; five were female and three were male, with a mean age of 71.5 years.

It was important to include participants with no prior experience with HAs as long-term fitted subjects would have had difficulty answering Question 10 of the SADL, which relates to amplification. Furthermore, participants with no prior experience of amplification could make a judgment based solely on the amplification fitted in this study.

**Table 6:** Summary of participant data

Name (abbreviation)	Gender	Age (years)	Distance between home and remote unit (miles/km)
RRS	Female	83	3/4.8
APS	Male	85	14/22.5
FRO	Male	73	7/11.2
MCS	Female	56	9/14.5
GPS	Male	90	3/4.8
RNS	Female	48	8/12.8
NLSN	Female	59	12/19.3
MEC	Female	79	9/14.5

### Design

We used an experimental research design. The following procedures were done face-to-face with the participants: (1) interview and otoscopy by otolaryngologist, (2) impedance and audiological measurements by audiologist, (3) agreement between patient and professional on the HA fitting, (4) earmold impressions, and (5) initial programming procedures with patient.

The following elements were remote (telefitting): (1) presence of an audiologist in RU, (2) presence of an audiologist in the SU (as the facilitator), (3) remote aid adjustments and changes to fitting data based on patient audiogram and subjective feedback, and (4) verification of patient satisfaction using the SADL questionnaire.

**Figure 2:**
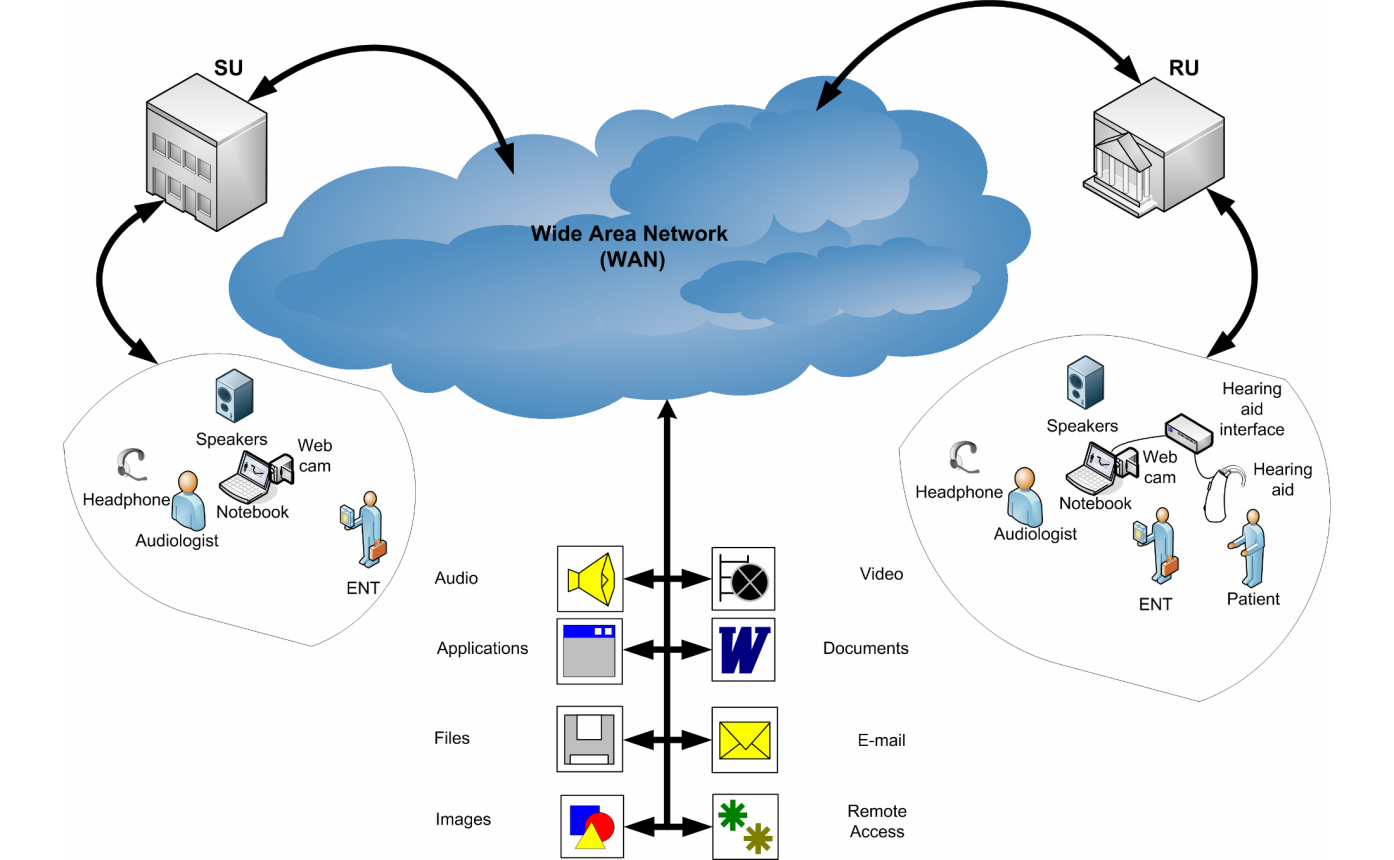
Basic scheme in telefitting session.

The SADL questionnaire was used as an interview schedule, that is, read aloud and completed by a trained interviewer for this purpose. The sessions were described as face-to-face (F) and remote assistance (R). There were two face-to-face sessions (F1 and F2) done within 15 working days of each other. The remote assistance (R) was 15 days after F2. This 15-day delay was justified so that patients would have adequate time and experience with the device and thus be able to respond accordingly to the SADL. At F1, patients agreed and signed the informed Consent Form (Chart 7). In the initial fitting sessions, the HAs were programmed through the easyFIT application, and the SU audiologist provided the necessary guidance to the patient. The face-to-face sessions followed the basic scheme described in Figure 1, while the remote service follows the basic scheme described in Figure 2.

The SU had an audiologist trained to fit the HA Mini Retro C through easyFIT while the audiologist at RU had no specific training for the Mini Retro C, nor for easyFIT. Both units were supervised by an otologist, while 2 information technology professionals offered the technological support, one for each side.

For the remote (telefitting) sessions (Figure 2), all patients were based at the RU and accompanied by the RU audiologist, who explained that the SU audiologist would not be physically present but would interact with the patient through the Internet. The HAs were removed from the ears of patients to check for the presence of wax in the earmolds and then reinserted by the RU audiologist. The programming cables were inserted into their HAs, thereby allowing the programming interface to read the connected HAs, with identified model and serial numbers as well as access to all patient information (data personal, audiometry, and data sessions, etc).

The SU audiologist then remotely accessed the fitting application easyFIT and began procedures to check the HA settings. The patients were questioned subjectively about various aspects of their HA usage, which allowed the audiologist at SU to make adjustments remotely. In all cases, adjustments were required. Remotely, the SU audiologist recorded the new settings in the HAs and updated patient information in easyFIT. Additionally, situational/environmental advice was provided to the patient remotely before the SU audiologist ended their interaction with the patient.

Finally, the RU audiologist administered the SADL. The methodology of the questionnaire was explained, and each question was read aloud. This allowed for participants with poor literacy to fully understand all questions posed. Frequent pauses and repetitions were allowed for so that the patient had time to think about the answers. The session ended with the signing of Terms of Donation (TD - Chart 7) of HAs, again read aloud, so that patients with poor literacy were made aware of issues around the need to service HA quarterly from the date of signing the TD, as well as warranty periods, etc.

## Results

In the initial telefitting session on September 18, 2012, 30 random measurements of Internet speed between the SU and RU were conducted (Table 7). Through the Datalogger feature for recording use of HAs, we found that over 50% (5/8) of patients used the HAs about 9 hours a day after being fitted, indicating good acclamation to the devices. Patient responses to the SADL questionnaire are presented in Table 8.

The results of the SADL from this study compared to four other studies are provided in Table 9 [43-45], which shows that the average scores from this study are above the mean scores from the original SADL normative data (Multimedia Appendix 3). These indicate a high level of satisfaction in participants who were fitted remotely.

**Table 7:** Internet speed in the specialized unit and the remote unit measured on September 18, 2012.

Parameters	RU			SU		
	Lowest	Highest	Average	Lowest	Highest	Average
Download^a^ (kbps^b^)	1521	4025	2842	9269	12,779	11,935
Upload^c^ (kbps)	552	2554	1820	7496	11,160	9910
Ping^d^ (ms^e^)	34.4	95.6	58.5	4.2	11.5	7.3

^a^Download: speed (kbps) to download a particular file server.

^b^kbps: kilobyte per second = 1000 bits/ second; the digital signal transmission rate.

^c^Upload: speed (kbps) to load a particular file server.

^d^Ping: latency; the time (ms) necessary to test connectivity between information technology devices.

^e^ms: millisecond = 0.001 second.

**Table 8:** Summary of responses of patients to the SADL^a^.

Patients	1	2	3	4	5	6	7	8	9	10	11	12	13
RRS	G	A	G	A	B	G	A	G	G	G	A	G	A
APS	G	A	G	A	G	G	A	G	G	G	G	G	A
FRO	G	A	F	A	G	G	A	F	G	F	F	G	F
MCS	G	F	G	B	E	G	A	G	G	G	D	G	A
GPS	G	A	G	G	G	G	A	G	G	E	E	G	A
RNS	G	A	G	A	G	G	A	G	G	E	G	G	A
NLSN	G	A	G	E	G	G	A	G	G	F	G	G	A
MEC	F	A	G	A	F	F	A	F	F	D	E	G	A

^a^A=not at all, B=a little, C=somewhat, D=medium, E=considerably, F=greatly, G=tremendously; see Multimedia Appendix 1.

**Table 9:** Results of our SADL compared with four other works (mean score and SD).

Factors	Cox and Alexander [9]	Danieli et al [43]	Mondelli et al [44]^a^	Farias and Russo [45]^a,b^	Our research (2012)
Positive effects	4.9 (1.3)	5.1 (1.3)	6.5 (0.5)	6.2 (0.8)	6.5 (0.4)
Negative features	3.6 (1.4)	4.5 (1.7)	6.3 (0.9)	6.2 (1.0)	6.2 (1.0)
Service and cost	4.7 (1.2)	5.5 (0.8)	4.7 (1.5)	6.7 (0.6)	7.0 (0.0)
Personal image	5.6 (1.1)	5.9 (0.9)	5.4 (1.6)	6.7 (0.4)	6.4 (0.7)
Average	4.7 (1.3)	5.2 (1.2)	5.7 (1.1)	6.4 (0.5)	6.5 (0.5)

^a^These authors presented a two-decimal precision of measurement, here rounded to only one decimal for purposes of comparison, according to National Center for Education Statistics NCES Standard: 5-3.

^b^These authors separated the results according to gender of patients. The results presented here are those of the larger group (males), although the gender difference is minimal.

## Discussion

### Principal Findings

Telefitting can help improve hearing health policies in Brazil by gradually expanding the current 139 accredited centers to centers closer to patients’ homes as Basic Services Units, or health clinics, so the patient can receive adjustments to their HAs in their homes, with greater comfort and a greater chance of success in hearing rehabilitation. The participants in our study had a mean age of 71.5 years and had to travel an average of 8 miles (12.8 km) from home to RU. Table 9 reflects the patient satisfaction index close to one (6.5) to the maximum (7.0) with a low standard deviation (0.5), which indicates promising data on perceived benefits of patients fitted remotely. However, when compared with the study by Alexander and Cox [9], there is a considerable gap: 4.7 (1.3). This occurs due to the idiosyncratic difference between the two distinct audiences: the audience described by Cox and Alexander is based on individuals who had recently purchased HAs with their own resources in the United States, while the target populations in this study were SUS patients who received donated HAs. It is evident that patients with their own resources had different expectations than the patients in this pilot study.

Despite missing official data, it is possible to speculate that all the government-run health centers have at least one PC and probably an Internet account, which highlights the possibility of performing telefitting supported by an SU. There are 537 Basic Services Units (ambulatory level) throughout the city of Sao Paulo alone. Furthermore, it may not be necessary to have the hardware component HI-PRO, since it could be replaced by an application installed over the Internet. A universal programming cable for all HA manufacturers could be used instead of standard cables for a particular manufacturer. In general, most clinics have to operate with a large number of programming cables for various manufacturers.

The 2010 Brazilian Census reports that between 2005 and 2008, Internet access increased by 75.3% or 56 million users, due to various factors such as PCs and notebooks as well as high-speed Internet connections being more accessible and available at a lower cost. These factors, combined with other applications, can promote the use of telemedicine. Swanepoel et al [46] have highlighted new innovative means of bringing hearing health services to people through the benefit of telemedicine. Wasowski et al [47] concluded that the Nationwide Network of Teleaudiology with cochlear implants was a reliable platform for telefitting.

In this study, applications and user-level information technology were used, which although limited, allowed for the fitting of HAs in 8 patients. Nevertheless, if more advanced technology were used (eg, application-specific videoconferencing), the possibility of conducting real-time orthoscopic reviews would be increased. In addition, traffic-encrypted data over the Internet, access from other accredited PCs on the network, as well as integration with other applications, would ensure that management costs are kept to a minimum. There would also be a reduced number of patient visits to the clinic and a database of valuable patient information to allow for remote HA adjustments.

In a more comprehensive telemedicine approach, it would be beneficial to include video training for the audiologist, as well as detailed training on the equipment and troubleshooting for complex fittings. An online tutor can assist in immediate cases (eg, when the audiologist at RU has doubts or is not familiar with fitting HAs), while full online courses on anatomy and physiology of hearing, interpreting audiometry, among others may be available.

Other questionnaires that could be used include the IOI-HA questionnaire, adapted to Brazilian Portuguese. It consists of seven questions, with a closed set of five different responses, and is thus easier to apply compared with SADL. The HHIE adapted to Brazilian Portuguese is structured into 25 questions, with a closed set of three possible answers on which Aiello et al [48] report that “further studies are needed to determine the convergent validity and construct validity of this instrument”. Finally, the APHAB, which has not been adapted to Brazilian Portuguese, has 25 questions, each of which has 14 possible levels (each question includes two scenarios: with and without HA).

One can standardize the application of a satisfaction questionnaire, after the initial HA fitting and before the end of the warranty period. Thus, two questionnaires could record satisfaction levels at two significant times in the hearing rehabilitation process.

### Strengths and Limitations

This study was one of the first of its kind with regard to adjusting HA settings via the Internet in Brazil. Furthermore, it creates a baseline for future research in this area of remote audiology and telefitting. However, our sample size of participants was small and within a limited geographical area. In addition, a limited number of applications were utilized.

### Recommendations

In this study, certain applications and features were used to perform remote adjustments and fitting of HAs in 8 patients without injury. In the more comprehensive telemedicine approach, it would be necessary for the RU audiologist to have additional training and support. Further studies with a larger sample population should be conducted to explore the reproducibility of the results recorded here.

For implementation and public policy, we recommend the Windows platform be replaced by an open platform (eg, Linux, Ubuntu, Android) in order to reduce costs and promote the development of local solutions. The Internet services should ideally be linked to attributes such as stability, availability, speed, absence of risk, and confidentiality of patient data must be protected.

Furthermore, investigations conducted with the Brazilian Portuguese version of IOI-HA and APHAB questionnaires may be valuable. Although recording patient satisfaction through questionnaires provides valuable information about the use of HAs, the information derived is not entirely sufficient to assess the quality of overall hearing health. Bevilacqua et al [49] stated that “assessing the quality of health services is based in the infrastructure”, that is, policies, facilities, and professionals. Silva and Formigli [50] generalized that in Brazil, the reorganization of health practices requires a definition of strategies for assessing changes of a broader management model.

A key step is to monitor satisfaction with technical issues by use of key performance indicators described by Kaplan and Norton [51], as a system of performance measurement and strategic management. As a result of understanding patients’ difficulties, there can be continuous improvement in public health.

### Conclusions

Remote HA adjustments (telefitting) have proved effective for these 8 patients, as indicated by their dynamic responses in SADL. Results were comparable to those of patients fitted in the conventional manner (ie, face-to-face fittings). Thus, the use of telefitting can be seen as an effective method to improve service delivery of hearing health in Brazil.

## Multimedia Appendix 1

Brazilian Portuguese version of the SADL.

## Multimedia Appendix 2

Mini Retro C Datasheet.

## Multimedia Appendix 3

The SADL normative data.

## Abbreviations

APHAB: Abbreviated Profile of Hearing Aid Benefit
BTE: behind the ear
HA: hearing aid
HHIE: Hearing Inventory for Elderly
IOI-HA: International Outcome Inventory for Hearing Aids
PC: personal computer
RU: remote unit
SADL: Satisfaction with Amplification in Daily Life
SU: specialized unit
SUS: Unified Health System
USB: universal serial bus
