# Supplementary material for: Use of the Satisfaction With Amplification in Daily Life Questionnaire to Assess Patient Satisfaction Following Remote Hearing Aid Adjustments (Telefitting)
Source: JMIR Med Inform. 2014 Sep 2;2(2):e18. doi: 10.2196/medinform.2769 (PMC4288118; doi:10.2196/medinform.2769)
Supplement: Supplementary file 1 [file medinform_v2i2e18_app1.pdf]

SATISFAÇÃO COM O APARELHO AUDITIVO EM SUA VIDA DIÁRIA

NOME ____

DATA DE NASCIMENTO __/___/____ **DATA** __/___/______

INSTRUÇÕES

Estão listadas abaixo, perguntas sobre o seu aparelho auditivo. Favor circular a letra correspondente a melhor resposta para você, para cada pergunta. A lista de palavras à direita lhe oferece o significado de cada letra.

A Nada
B Um pouco
C De alguma forma
D Mediamente
E Consideravelmente
F Muito
G Muitíssimo

Lembre-se que suas respostas devem mostrar suas opiniões gerais em relação ao aparelho auditivo que você está usando agora ou aquele que utilizou mais recentemente.

1- Seus aparelhos auditivos lhe ajudam a entender o que as pessoas que conversam mais freqüentemente com você falam, quando comparado sem o uso dos aparelhos?	**A**	**B**	**C**	**D**	**E**	**F**	**G**
2- Você fica frustrado quando o seu aparelho capta sons que não permitem que você ouça os sons que gostaria de ouvir?	**A**	**B**	**C**	**D**	**E**	**F**	**G**
3- Você está convencido de que adquirir os seu aparelhos foi sua melhor opção?	**A**	**B**	**C**	**D**	**E**	**F**	**G**
4- Você acha que as pessoas percebem mais a sua perda aditiva quando você está usando o seu aparelho auditivo?	**A**	**B**	**C**	**D**	**E**	**F**	**G**
5- Os seus aparelhos reduzem o número de vezes que você tem que pedir para as pessoas repetirem o que disseram?	**A**	**B**	**C**	**D**	**E**	**F**	**G**
6- Você acha que o seu aparelho compensa seu problema?	**A**	**B**	**C**	**D**	**E**	**F**	**G**
7- Você está chateado por não conseguir ter o volume que deseja sem que o aparelho apite?	**A**	**B**	**C**	**D**	**E**	**F**	**G**
8- O quanto você está satisfeito com a aparência de seus aparelhos?	**A**	**B**	**C**	**D**	**E**	**F**	**G**
9- Usar o aparelho melhora a sua autoconfiança?	**A**	**B**	**C**	**D**	**E**	**F**	**G**

A Nada
B Um pouco
C De alguma forma
D Mediamente
E Consideravelmente
F Muito
G Muitíssimo

10- Quão natural é o som que recebe de seu aparelho?	**A**	**B**	**C**	**D**	**E**	**F**	**G**
11- O quanto seus aparelhos ajudam ao falar em telefones que não tenham amplificadores de volume? (Se você escuta bem ao telefone ***sem*** os aparelhos selecione aqui ❍)	**A**	**B**	**C**	**D**	**E**	**F**	**G**
12- Quão competente era a pessoa que lhe forneceu os aparelhos?	**A**	**B**	**C**	**D**	**E**	**F**	**G**
13- Você acha que uzar o aparelho faz você se sentir menos capaz?	**A**	**B**	**C**	**D**	**E**	**F**	**G**
14- O custo do seu aparelho lhe parece razoável?	**A**	**B**	**C**	**D**	**E**	**F**	**G**
15- Você está satisfeito com a qualidade do seu aparelho (com relação ao número de vezes que ele precisou de reparo)?	**A**	**B**	**C**	**D**	**E**	**F**	**G**
