# Supplementary figures and images for: Use of the Satisfaction With Amplification in Daily Life Questionnaire to Assess Patient Satisfaction Following Remote Hearing Aid Adjustments (Telefitting)
Source: JMIR Med Inform. 2014 Sep 2;2(2):e18. doi: 10.2196/medinform.2769 (PMC4288118; doi:10.2196/medinform.2769)

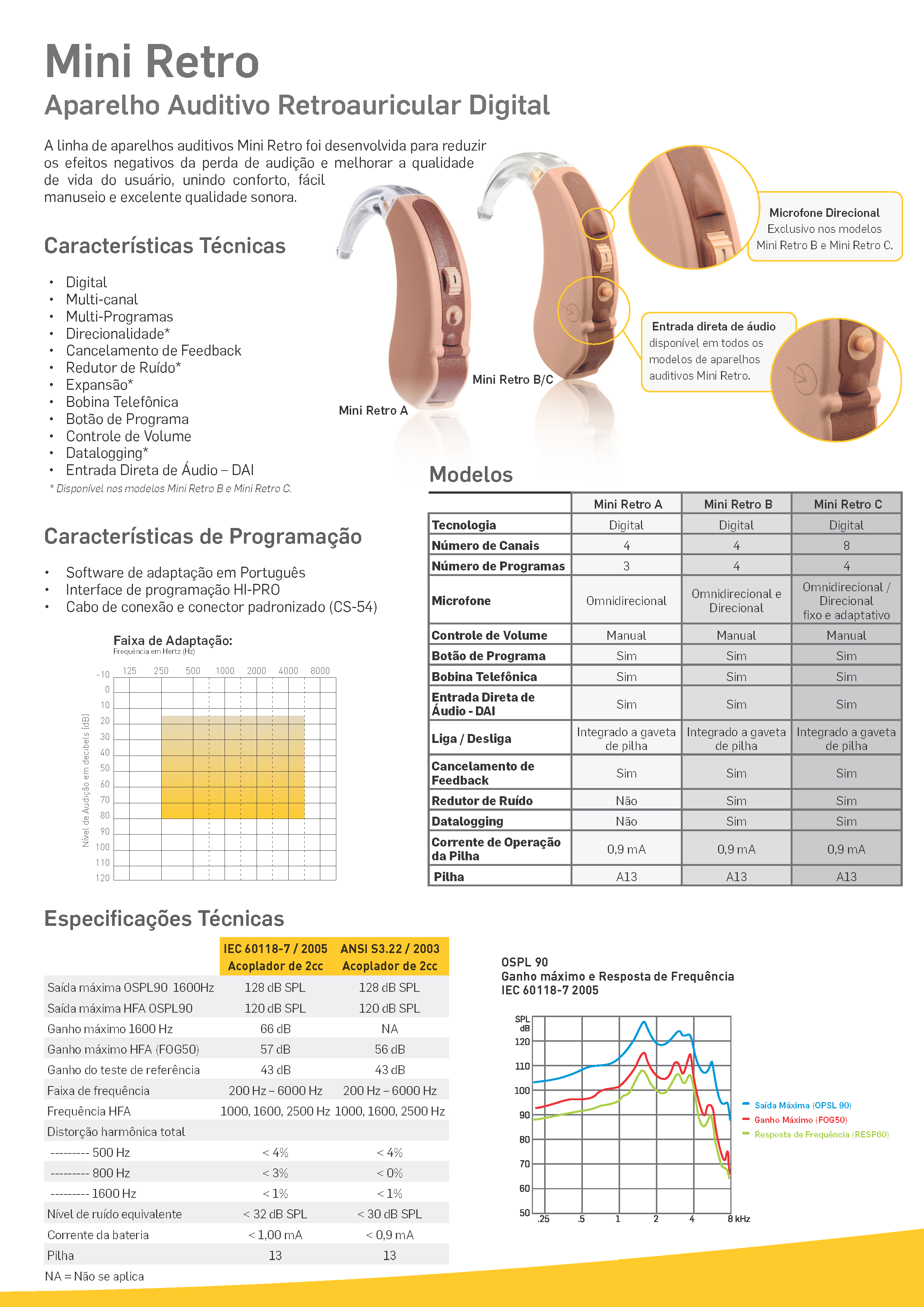

Supplement: Supplementary file 2 [file medinform_v2i2e18_app2.jpg]

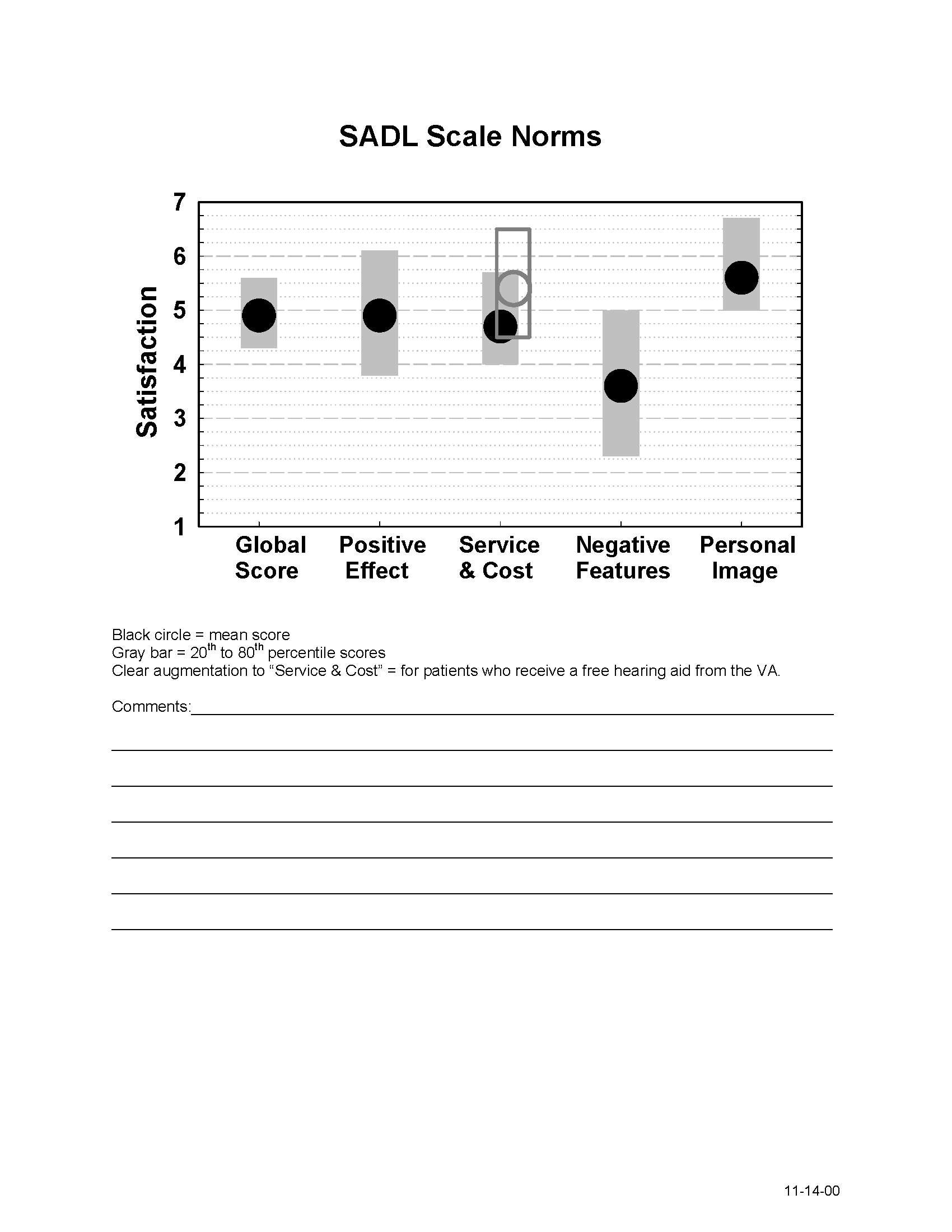

Supplement: Supplementary file 3 [file medinform_v2i2e18_app3.jpg]
